# Performance bonuses in the public sector: Winner-take-all prizes versus proportional payments to reduce child malnutrition in India

**DOI:** 10.1016/j.jdeveco.2018.10.003

**Published:** 2020-09

**Authors:** Prakarsh Singh, William A. Masters

**Affiliations:** a Research Affiliate, Institute of Labor Economics (IZA), Germany; b Tufts University, Friedman School of Nutrition and Department of Economics, 150 Harrison Ave., Boston, MA 02111, USA

**Keywords:** Contest design, Performance pay, Malnutrition, Underweight, Child development

## Abstract

We conduct a randomized trial to compare incentives for improved child outcomes among salaried caregivers in Chandigarh, India. A contest whose prize is divided among workers in proportion to measured gains yielded more improvement than a winner-take-all program. In our population of about 2000 children served by 85 workers, using proportional rewards led to weight-for-age malnutrition rates that were 4.3 percentage points lower at 3 months (when rewards were paid) and 5.9 points lower at 6 months (after the contest had ended), with mean weight-for-age z scores that were 0.071 higher at 3 months, and 0.095 higher at 6 months. Proportional bonuses led to larger and more sustained gains because of better performance by lower-ranked workers, whose efforts were not rewarded by a winner-take-all prize. Results are consistent with previous laboratory trials and athletic events, demonstrating the value of proportional rewards to improve development outcomes.

## Highlights

• Bonuses can be paid in proportion to success, instead of winner-take-all (WTA) prizes.
• An RCT with caregivers in India found more gains from proportional bonuses than WTA.
• Weight-for-age malnutrition rates (WAZ < −2) were 5.9 points lower after 6 months.
• Proportional bonuses do better by eliciting more gains among lower-ranked workers.
• Employers may prefer to reward improvement by all workers, not just top performers.

## 1 Introduction and motivation

Prize contests and other performance incentives offer a promising approach to improving public service provision. Teachers, nurses and other service providers often have limited information about the success of their efforts, and existing arrangements may not reward them for significant achievements. Performance pay and bonuses can help align worker interests with beneficiary needs, and reveal information about how to improve in fields such as education (Muralidharan and Sundararaman, 2011; Behrman et al., 2015) and health (Scott et al., 2011; Miller and Babiarz, 2014).

This study reports on a randomized trial of incentives offered to Anganwadi workers serving preschool children in daycare centers across the urban slums of Chandigarh, India, as part of the government's Integrated Child Development Services (ICDS) program. Each Anganwadi worker manages her own ICDS center, typically a single room, in which she is expected to provide a mid-day meal, daycare and some educational services for about 25 children from 3 to 6 years of age. A principal objective of the ICDS program is to help children avoid and recover from malnutrition, defined by the government of India as low weight for age, by complementing whatever food and care is provided by each child's own family. Workers are salaried civil servants, for whom disciplinary actions in the case of poor performance are generally ineffective (Verma et al., 2018), leading some states to introduce positive incentives for better outcomes (Kahn, 2017).

The incentives we offer aim to identify how ICDS managers can best recognize and reward Anganwadi workers for their otherwise neglected efforts, building on a series of previous experiments in Chandigarh and elsewhere (Singh, 2015; Singh and Masters, 2017; Singh and Mitra, 2017). The specific study presented here compares two canonical types of incentives offered in addition to the workers' base salaries: winner-take-all contests in which workers compete for a fixed prize, and proportional rewards in which that amount is divided among workers in proportion to their share of total achievement. With winner-take-all prizes, reward is based on rank order and most workers receive nothing, whereas with proportional payments every increment of success is always rewarded and all competitors may receive something for even small improvements in performance. Our design aims to identify differences in how workers respond to the two types of incentives during the contest period when these rewards are offered, and also after they are withdrawn.

A key feature of our trial is the use of identical information and payment budgets in the two treatment arms, so any differences in response are attributable to the way funds are distributed. This design complements a previous trial in this setting which compared piece-rate payments to a fixed bonus and a control arm in which workers received only their base salary (Singh and Masters, 2017). Those treatments all used the same information, but differed in amounts of money paid. Other trials differ in many dimensions, as in the introduction of new reward schemes relative to the status quo without performance incentives (Banerjee et al., 2010), or the comparison of financial versus symbolic prizes (Ashraf et al., 2014), fixed budgets versus payment for services delivered (Basinga et al., 2011; De Walque et al., 2015), or outputs rather than inputs (Mohanan et al., 2017).

Our trial is designed to inform how service-delivery and development organizations use contest incentives, especially in field settings like the ICDS in India where links between workers' efforts and outcomes are not clear, and where social norms or intrinsic motivations play an important role. Philanthropists and public agencies who introduce new incentives typically choose winner-take-all prizes (McKinsey, 2009), in part because both laboratory experiments and field data suggest that these contests often elicit the most effort by top-ranked contestants. Rank-order competition offers “high-powered” incentives, concentrating all of the available reward at the margin between the best and next-best achievement levels (Lazear and Rosen, 1981). Winner-take-all prizes also activate behavioral motivations associated with competition itself, as many participants respond to rank-order contests with more effort than the discounted expected value of whatever material rewards are actually offered (Dechenaux et al., 2015). Using such prizes may not be appropriate, however, in situations where links from effort to outcome is unclear, or there is great variation in the difficulty of each task relative to workers' ability. Cason, Masters and Sheremeta (2010) used a laboratory experiment to show that workers whose initial experience is less successful tend to withdraw from competition, thus reducing total effort expended. Brown (2011) found a similar result in real-world athletic competitions, where the presence of a superstar who reduces payoffs to others leads them to reduce their efforts.

The discouragement effect that may be associated with winner-take-all contests could potentially be overcome using proportional incentives, paying bonuses to all workers in proportion to their success. Organizations like the ICDS might want to pay incentives in proportion to success partly to elicit more effort from even the lower-performing workers, but also to avoid the displacement of intrinsic motivations and social norms that is associated with winner-take-all contests. As shown by Cason et al. (2018), the higher efforts elicited by winner-take-all prizes among top competitors occur at the expense of total welfare for the sum of all workers, while the more moderate efforts exerted under proportional rewards are closer to Nash equilibrium levels and hence less likely to be regretted after the contest ends. Lazear (2000) finds that a shift from fixed wages to piece-rate pay raised effort and productivity in a manufacturing setting, demonstrating the value of introducing some kind of output-based reward, but Bandiera et al. (2005) find that a further switch from piece rates to a relative pay scheme, in which individual effort imposes a negative externality on peers by lowering the other's relative performance, led to a subsequent productivity decrease. Foster and Rosenzweig (1994) find evidence of moral hazard in effort by farm laborers, Amodio and Martinez-Carrasco (2018) find evidence of shirking from group-based incentives, and in a more extreme case, Chen (2003) finds evidence that winner-take-all rewards encourage destructive sabotage among competitors. Proportional payments at the individual level could help align incentives by rewarding each person's efforts more equally, revealing what works and reinforcing norms that could improve outcomes even after rewards are withdrawn.

## 2 Experimental design

Our intervention was implemented in collaboration with the Social Welfare Department of Chandigarh, aiming for three specific contributions to the literature on incentives for public sector service delivery in the context of a developing country:

First, we compare winner-take-all (WTA) prizes with proportional reward payments (PRP) in a fully controlled randomized trial, where each treatment uses the same information and involves the same fixed level of budgeted expenditure. The only difference is in the distribution of funds. Putting the same information and financial resources into each arm helps overcome the problem that previous trials often combine multiple features in ways that preclude isolating the effect of any one aspect of program design (e.g. Christianson et al., 2008; Eichler et al., 2009). Total funds available are identical which limits differences in overall income effects, and both schemes impose the same potential total cost to the sponsor which is known in advance and facilitates budgeting.

Second, we compare differences over two rounds of outcome measurement three months apart (henceforth, Endline-1 and Endline-2), to test for longer-term persistence of effects after incentives are withdrawn. This is particularly important given concerns that introducing competition could change habits or norms, displacing workers' intrinsic motivations and altering social relationships as in the framework described by Franco et al. (2002).

Third, our incentives are paid for health outcomes, like Gertler and Vermeersch (2012) and Miller et al. (2012) rather than service provided like Bhushan et al. (2007). We also conduct mechanism checks on what workers actually did to alter those outcomes and their self-reported level of satisfaction with their work. If one treatment arm was more successful than the other, knowing how those workers achieved that change could help scale up success elsewhere, and knowing if worker satisfaction improved or worsened is important for personnel management. Incentive schemes that target outcomes and also sustain job satisfaction are difficult to design, as shown in previous studies of village health workers (Amare, 2009; Bhattacharyya et al., 2011; Speizer et al., 2001), government agencies (Hasnain et al., 2014) and non-governmental organizations (Loevinsohn and Harding, 2005).

The incentives we offer target the primary nutritional outcome of interest to ICDS management, which is the number of children classified as malnourished by their weight for age. This type of malnutrition is defined by the World Health Organization as a weight-for-age z score (WAZ) that is more than 2 standard deviations below the median of a sex-specific healthy population. To inform Anganwadi workers and ICDS managers about their progress towards that objective, we created goal cards for each child, showing their current weight and, if malnourished, the target weight they would need to achieve to be classified as no longer malnourished when re-weighed three months later. For normal weight children, a threshold weight was specified below which the child would be classified as malnourished when weighed three months hence, with any such declines in nutritional status counted against any gains. This symmetry limits the degree to which workers are incentivized to help only malnourished children at the expense of the healthier children, while maintaining the ICDS management's focus on the fraction of children above the WAZ = −2 threshold.

The trial design features randomization at the level of individual workers within neighborhoods. This ensures that workers have common information, similar ICDS management and other conditions. We enrolled a total of 85 workers serving a total of about 2200 children and their mothers, located in 6 slum areas outside central Chandigarh. Each worker reports to a supervisor who is in charge of one neighborhood. We randomly assigned individual workers in each neighborhood to one of the two treatment arms, and offered payments based on the total number of children in their center whose nutritional status improved, relative to the improvements achieved by other workers in their neighborhood who drew the same treatment. Our focus on the number of children who cross this malnutrition threshold is dictated by ICDS policy, and facilitated communication about the contest. Counting only the prevalence of weights above the threshold could lead workers to neglect children who are far from the thresholds, however, so future contests could use net gains in a continuous measure of nutritional status, such as average distance from a target weight. Future incentives could also take account of other child development objectives, such as attendance and learning.

**Fig. 1:**
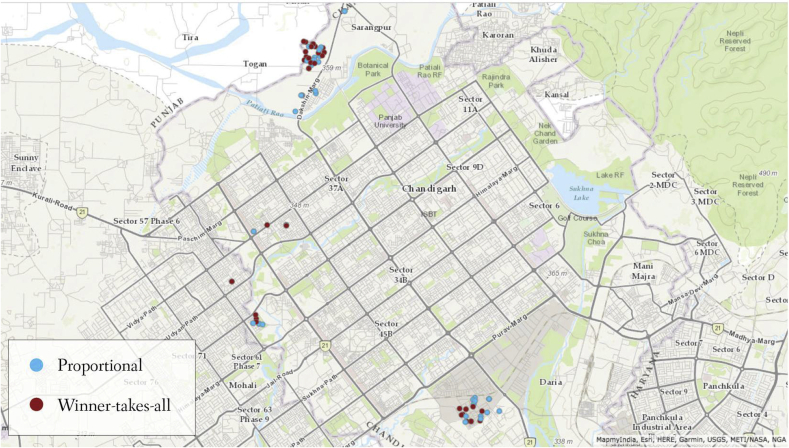
Map of Anganwadi centers in Chandigarh, by treatment.

Fig. 1 shows the geographical location of all centers and the treatment to which they were assigned. Neighborhood boundaries are not shown, but the map clearly reveals the close proximity among centers in the most densely populated slums. Randomized assignments were carried out through lottery in the presence of other workers from each neighborhood, to ensure transparency among workers and their supervisors. Rewards were paid out in similar gatherings after three months. This approach implies that any effects we observe for PRP relative to WTA arise among workers who know that the other type of incentive exists. To compare effects of contest design among naïve workers would require cluster randomization across isolated groups, and even then workers in groups who all receive either PRP or WTA incentives could readily infer that the same funds might be paid out in different ways.

Timeline and implementation involved four rounds of data collection at three-month intervals. Child weights are the basis of the intervention and also the primary outcome of interest. Each round included weighing all children at every center, and also interviewing their mothers, using trained survey staff from the private sector. We also obtained the ICDS system's own administrative data on all children enrolled in each center, their mothers, and the workers, and conducted a separate survey of workers at the end of the experiment. A first round of data collection in October 2014 (Baseline-1) was used familiarize respondents with the data-collection process, and then a second round in January 2015 (Baseline-2) was used to construct the goal cards for each worker, which we distributed to caregivers in early February along with their random assignment to either WTA or PRP incentives. Children were weighed again in April 2015 (Endline-1) to compute payouts for each worker, which was followed immediately in early May by actual payment of prizes to winners in the WTA arm, and proportional rewards to workers with net gains in the PRP arm. A final round in July 2015 (Endline-2) allowed us to test for persistence of any treatment effects, and at that same time we also collected data on worker satisfaction given the outcome of the trial. A visual summary of this sequence is provided in the annex of supplemental information (Fig. A1).

Payments in the WTA treatment follow equation (1), in which worker *i* in cluster *k* is compared in terms of the number of improvements *n* she achieves relative to any other worker *j* in her treatment group. The highest performer in each cluster receives the entire bonus amount available for that group:

(1) $$WTA_{ik}=\{\begin{array}{c} B_{k}\;if\;n_{i}>n_{j}\;for\;all\;j\;in\;k\\ 0\;if\;n_{i}<n_{j\;}\;for\;any\;j\;in\;k \end{array}$$

Payments in the PRP treatment follow equation (2), in which worker *i* in cluster *k* receives a share of the amount available for her cluster that equals her share of all improvements achieved by all workers in her group:

(2) $$PRP_{ik}=\{\begin{array}{c} \frac{n_{i}B_{k}}{\sum_{j=1}^{N}n_{j}}\;\\ 0\;if\;n_{i}<0 \end{array}$$

The workers were told that if a normal-weight child fell below their threshold weight, that decline would be subtracted from the number of children whose status improved. Payments would be based in the net number of improvements, n, recorded among the children attending the worker's center. Payments in both arms are truncated at zero, so workers cannot lose money by participating in the trial. Groups in which no workers achieved net improvements would have no payouts, and in the event of a tie among workers in the WTA treatment, two or more workers could share the prize equally. In the PRP arm, all workers who achieve some improvement receive their proportional share of the group's entire bonus.

The available bonus pool for each treatment group was set at 600 Rs per worker. The level of payment is designed to be just sufficient to elicit workers' effort based on Singh and Mitra (2017), so as to achieve the highest possible level of cost-effectiveness. Using bonus pools with a fixed budget per worker also facilitates replication across places with varying number of workers. Group size alters the payoff formula in WTA, as each worker in larger groups has a lower probability of winning a larger prize, but in PRP the payoff is less susceptible to group size as workers in larger groups can expect to receive a smaller share of a larger reward.

In the neighborhoods selected for our trial, the smallest treatment area had only three workers. The minimum size for a contest is two workers, so this area could not be split between two arms, and by random draw all three workers were assigned to the WTA contest. Two neighborhoods had four centers and were split evenly with two workers in each arm. One neighborhood had seven centers and drew three in the WTA arm and four in the PRP arm. Another had 29 centers, drawing 14 for WTA and 15 for the PRP treatment, and one had 38 centers drawing 19 in each arm. The names of each neighborhood are detailed in the annex of supplemental information, showing the number of workers in each treatment group (Table A1).

Actual payments were based on changes in measured weights after 3 months. The number of children measured in each group at that time is listed in the annex of supplemental information (Table A2.1). A total of 1225 children at 43 centers in six neighborhoods were measured in the WTA arm, and 1115 children at 42 centers in five neighborhoods were measured in the PRP arm. As it happened, net gains in malnutrition status occurred in only three of the six WTA groups, so only three of the 43 workers in that arm received a payout. Those prizes averaged 4000 Rs, about one month's salary for these workers. In the PRP arm, net gains occurred in all five neighborhood groups, at centers managed by 16 of the 42 workers. All 16 of them received a payout, averaging 1575 Rs each. Payouts are listed and shown graphically in the annex of supplemental information (Table A3 and Fig. A4).

## 3 Data

Table 1 shows summary statistics from the Baseline-2 survey when randomization occurred. Column 3 shows the differences between the two arms along with their adjusted standard errors. None of the characteristics have statistically significant differences, so randomization between the arms was successful in these terms. Testing for treatment effects can proceed directly, but for completeness our tests include results with statistical controls for child, mother and worker characteristics, and a placebo test for artefactual effects prior to randomization and treatment. All of the child regressions have standard errors clustered at the level of the ICDS center.

**Table 1:** Summary statistics at baseline in winner-take-all (WTA) and proportional payment (PRP) treatment arms.

	WTA (1)	PRP (2)	Difference (3)		WTA (4)	PRP (5)	Difference (6)
**Demographic characteristics**				**Child diet (proportion consuming each food at least twice per week)**			
Child age	4.58 (0.989)	4.56 (0.958)	−0.0215 (0.0554)	Milk	1.00 (0.0643)	0.99 (0.102)	−0.00626 (0.00666)
Child is male	0.50 (0.500)	0.51 (0.500)	0.00944 (0.0154)	Dal	0.96 (0.200)	0.95 (0.228)	−0.0132 (0.0209)
Mother age	27.17 (3.925)	27.20 (3.899)	0.0294 (0.239)	Green Veg	0.60 (0.489)	0.56 (0.497)	−0.0444 (0.102)
Number of children	2.35 (1.362)	2.29 (1.401)	−0.0539 (0.128)	Fruit	0.48 (0.500)	0.49 (0.500)	0.0138 (0.0976)
Household income	8639.44 (3330.6)	8379.58 (2765.6)	−259.9 (378.0)	Sweets	0.13 (0.333)	0.23 (0.420)	0.101 (0.0646)
Mother illiterate	0.38 (0.486)	0.36 (0.481)	−0.0163 (0.0366)	Egg	0.25 (0.434)	0.27 (0.444)	0.0173 (0.0599)
Worker age	37.70 (7.969)	38.95 (8.161)	1.252 (1.769)	Dalia	0.89 (0.319)	0.88 (0.320)	−0.00113 (0.0368)
Worker college	0.43 (0.496)	0.45 (0.497)	0.0119 (0.110)	Chicken	0.11 (0.313)	0.13 (0.338)	0.0209 (0.0410)
**Child weight outcomes**				Roti	0.92 (0.272)	0.94 (0.239)	0.0196 (0.0223)
Weight for Age (WAZ)	−1.54 (0.808)	−1.48 (0.771)	0.0552 (0.0668)				
				Chips	0.43 (0.495)	0.38 (0.486)	−0.0491 (0.0792)
Malnourished (WAZ < −2)	0.27 (0.446)	0.28 (0.449)	0.00545 (0.0321)				
Weight (kg)	13.73 (1.885)	13.81 (1.885)	0.0719 (0.148)	**Worker effort (number per month, as reported by mother)**			
				Visits the home	7.61 (6.155)	6.41 (4.794)	−1.194 (1.066)
**Mother nutrition knowledge**							
Quiz 1 (18 questions)	10.72 (2.890)	11.10 (2.597)	0.377 (0.449)	Talks about child	10.62 (8.571)	11.26 (8.472)	0.639 (1.780)
Quiz 2 (25 questions)	15.21 (3.312)	15.76 (3.162)	0.558 (0.533)	Hosts meeting at AWC	4.29 (3.106)	3.73 (1.696)	−0.561 (0.391)
Quiz 3 (7 questions)	4.48 (1.209)	4.66 (1.228)	0.181 (0.224)	Has other meetings	8.32 (6.205)	9.35 (6.696)	1.037 (1.280)

Note: Data shown are mean and standard deviations, with standard errors and t-tests for significance of differences. Differences are not statistically significant (p = 0.5). All data shown here are measured at Baseline II (January 2015). Sample size is 43 workers with 1022 children and mothers in the winner-take-all (WTA) arm, and 42 workers with 1180 children and mothers in the proportional payment (PRP) arm.

The summary statistics in Table 1 reveal key features of the ICDS system, including a large gap in age and education between children's mothers (averaging 27 years of age with about 37% illiteracy) and the Anganwadi caregivers (averaging 38 years with about 44% identifying as college educated). Child malnutrition is widespread, with the average weight-for-age z score around −1.5 standard deviations below the median for a healthy child of their age and sex, and 28% of the children officially classified as malnourished by that standard (z  <  −2) as defined by the World Health Organization (WHO). Child diets most often include milk, ‘dal’ (lentil), ‘dalia’ (porridge) and roti (wheat bread), with less frequent consumption of green vegetables, chips and fruit, and less common intake of fruit, eggs or chicken. Worker effort is highly variable, with standard deviations almost as large as the means for how often mothers say the worker visits their home, talks about the child, hosts a meeting of mothers at the Anganwadi center, or has other meetings with the mother.

Repeated measurements of child weight are vulnerable to heaping and serially correlated errors, as illustrated by diagnostic figures in the annex of supplemental information. First, histograms of weights and ages at Baseline-2 reveal considerable heaping in child weight at integer values from 10 to 15 kg (Fig. A2). Second, a scatter plot of changes in weight-for-age z scores from Baseline-2 to Endline-1 reveals mean reversion, as children who are initially more underweight experience larger gains, and those with higher initial weight-for-age experience more decline (Fig. A3). A pattern of this type could be due to biological or behavioral responses to weight change, but could also be driven by measurement error between the two surveys. Heaping and random errors would attenuate estimated treatment effects, but not threaten the validity of our experimental design.

## 4 Hypothesis tests

The empirical specification for our main results is:

(3) $$W_{ijt}=\beta {\left(proportional\right)}_{ijt}+X_{ijt}+C_{jt}+\varepsilon_{ijt}$$

Our treatment variable of interest, *proportional*, takes the value 1 if the worker is assigned to the payment bonuses for their share of all children whose status improves and zero otherwise, so β is the average treatment effect of this relative to a winner-take-all prize. The outcome variable W is a continuous measure of child weight, in kilograms or as a z score of weight-for-age, and the subscript *i* represents the individual child, the subscript *j* represents the Anganwadi center, and the subscript *t* is the survey round. We use the same structure with W as the ICDS management objective itself, which is an indicator equal to 1 if the child is classified as malnourished. Other tests include mechanism checks for heterogeneity by baseline anthropometric status, mechanism checks on worker effort as reported by the mother, and tests for differences in self-reported worker satisfaction after completion of the trial. Mechanism checks for the longer-term effects include interacting the treatment with the level of payout in the short-term, to test the impact of realized outcomes on persistent treatment effects.

Each test is conducted both with and without the *Χ*_*ijt*_ matrix of mother and child controls, and the *C*_*jt*_ matrix of center-level variables. Child and mother-level controls include the sex and age in months of the child, the age of the mother, the number of children in the home, the total household income, and if the mother can read and write. The worker-level controls include the Anganwadi worker age and if the Anganwadi worker is college educated. Errors are represented by *ε*_*ijt*_ and are clustered at the Anganwadi level. Attrition from one round to the next is a significant concern, as Anganwadi centers routinely have a high level of churn as children are often absent and may return or transfer to another school. Annex Tables A2.2 and A2.3 show that about 75–80 percent of the children weighed at baseline are re-weighed at the endlines, but there are no significant differences in attriters between treatment and control arms.

## 5 Main results: child weight

Table 2 shows the principal result of our trial, which is an increase in weight-for-age z scores and a reduced prevalence of weight-for-age malnutrition when workers are offered bonuses in proportion to success rather than through a winner-take-all prize. Panel A shows the short-term results for which incentives are paid after 3 months, while Panel B shows long-term results 3 months later after the incentives have ended. Magnitudes are greater in the longer term, with an estimated average treatment effect on malnutrition prevalence after 3 months of 4.3 percentage points and then 5.9 percentage points after 6 months. Precision of the estimate also increases over time, with treatment effect significant after 3 months at the 0.10 level that holds only in the unconditional test (columns 2 and 3), but after 6 months that significance holds even when staggering in controls for child, mother and worker characteristics (columns 5–6 and 8–9), including when outcomes are measured as raw weights (columns 4 and 7), and holds at the 0.05 level for the prevalence of malnutrition (column 3). We also tested for treatment effects as in columns 1–3 but controlling only for child age, and results were unchanged. Results in Appendix Table A5 show that the long-term impact was greater and more significant across all specifications. The proportional treatment's impact was twice as high after six months than after three months, for both weight and weight-for-age z scores. Thus, even after controlling for selection effects by restricting regressions to the same number of children across all rounds and specifications, we see the higher coefficients in Panel B providing supporting evidence for the explanation in Table 2. Randomization occurred within neighborhoods, so our regressions are run at the child level. Adding neighborhood fixed effects would leave the estimate using weight-for-age z scores insignificant at the 10% level, with a p-value of 0.138, but not alter the basic results for long term impacts on weight and weight-for-age malnutrition. To address sample size concerns we also conduct randomization inference using the -ritest- command of Hess (2017), and find no change in results.

**Table 2:** Effects on child weight of proportional payments vs. winner-take-all prizes, after 3 and 6 months.

	Weight (1)	Wfa z (2)	Wfa mal (3)	Weight (4)	Wfa z (5)	Wfa mal (6)	Weight (7)	Wfa z (8)	Wfa mal (9)
**Panel A: Short-term (Endline I, after 3 months)**									
Proportional	0.0764 (0.107)	0.0705* (0.0417)	−0.0432* (0.0256)	0.138 (0.0854)	0.0638 (0.0406)	−0.0381 (0.0248)	0.138 (0.0854)	0.0642 (0.0407)	−0.0369 (0.0244)
Child and mother-level controls				X	X	X	X	X	X
Worker Controls							X	X	X
N	2348	2342	2342	1665	1665	1665	1665	1665	1665
Adj. R-sq	−0.000	0.002	0.002	0.336	0.037	0.008	0.335	0.036	0.007
**Panel B: Long-term (Endline II, after 6 months)**									
Proportional	0.202 (0.122)	0.0951* (0.0504)	−0.0589** (0.0288)	0.209** (0.104)	0.0875* (0.0500)	−0.0517* (0.0280)	0.200* (0.101)	0.0824* (0.0482)	−0.0496* (0.0279)
Child and mother-level controls				X	X	X	X	X	X
Worker Controls							X	X	X
N	2325	2272	2272	1935	1934	1934	1935	1934	1934
Adj. R-sq	0.002	0.003	0.004	0.342	0.035	0.015	0.345	0.041	0.016

Notes: Dependent variables are the child's weight in kilograms (columns 1, 4 and 7), weight-for-age z score (columns 2, 5 and 8), and weight-for-age malnutrition status (columns 3, 6 and 9) defined as an indicator equal to 1 if weight-for-age z score <2. Panel A uses outcomes at Endline I in April 2015, for which payouts were made in May 2015. Panel B uses outcomes at Endline II in July 2015, several months after the contest ended. "Proportional" is an indicator equal to 1 for children at centers randomized into proportional rewards, relative to the omitted control which is a winner-take-all prize. Child and mother-level controls include age and sex of the child, the age of the mother, the total number of children in the home, the total household income, and if the mother cannot read and write. Worker-level controls include the Anganwadi worker's age and if she is college-educated. Heteroscedasticity-consistent standard errors accounting for clustering at the Anganwadi center level in parentheses. *Significant at 10%, **Significant at 5%, *** Significant at 1%.

The rise over time in magnitude and significance of effect sizes is a striking feature of our results. Contest design has a greater effect on outcomes after the competition has ended, when child weights no longer affect workers' financial compensation. This could arise simply because actions taken by workers during the contest to raise child weights have increasing impacts over time, but could also arise because contest design has lasting effects on workers' intrinsic motivations and social norms. Of course it is also possible that significant treatment effects at Endline-2 are actually an artifact of the experiment. Randomization after Baseline-2 may have failed in a subtle way, despite being balanced in terms of observables as shown in Table 1. To check robustness we conduct additional falsification tests, using children's weights prior to treatment as a placebo outcome. This reveals no evidence of artefactual treatment effects, as shown in the annex of supplemental information (Table A4).

Table 3 addresses the mechanism of impact by testing for heterogeneity in effects among Anganwadi workers by their level of performance. Winner-take-all awards are highly powered in the sense that they concentrate all their resources on the top performers, while proportional incentives reward every increment of improvement including gains among poor performers. If WTA works best among top performers, then the average treatment effect of PRP that is observed in Table 2 must have worked primarily by raising outcomes among the below-average performers. Table 3 tests this prediction by interacting our treatment variable with the worker's relative performance as defined by equation (2), which is the formula used to compute payouts in the proportional arm.

**Table 3:** Mechanism check: heterogeneity of treatment effects after 3 and 6 months, by level of payout.

	Wfa (z score)		Weight (kg)		Wfa (z score)		Weight (kg)		Wfa (z score)		Weight (kg)	
	3 mo. (1)	6 mo. (2)	3 mo. (3)	6 mo. (4)	3 mo. (5)	6 mo. (6)	3 mo. (7)	6 mo. (8)	3 mo. (9)	6 mo. (10)	3 mo. (11)	6 mo. (12)
Proportional	0.0703* (0.0416)	0.0947* (0.0496)	0.0760 (0.107)	0.203* (0.121)	0.0638 (0.0406)	0.0880* (0.0497)	0.138 (0.0854)	0.210** (0.104)	0.0642 (0.0408)	0.0836* (0.0481)	0.138 (0.0858)	0.202** (0.100)

Difference to mean payout of group (×1000)	0.0111 (0.0129)	0.0351** (0.0144)	0.0256 (0.0215)	0.0403 (0.0280)	0.0181 (0.0126)	0.0307** (0.0122)	0.0342 (0.0273)	0.0667** (0.0258)	0.0180 (0.0122)	0.0278** (0.0123)	0.0341 (0.0268)	0.0610** (0.0255)

Difference to mean payout*Proportional (×1000)	−0.0101 (0.0146)	−0.0508*** (0.0170)	−0.0376 (0.0269)	−0.0935** (0.0358)	−0.0158 (0.0140)	−0.0447*** (0.0139)	−0.0317 (0.0300)	−0.0920*** (0.0293)	−0.0163 (0.0145)	−0.0377*** (0.0140)	−0.0316 (0.00310)	−0.0783*** (0.0289)

Child and mother-level controls					X	X	X	X	X	X	X	X
Worker Controls									X	X	X	X

N	2342	2272	2348	2325	1665	1934	1665	1935	1665	1934	1665	1935
**adj. R-sq**	0.001	0.005	−0.001	0.003	0.036	0.036	0.335	0.342	0.035	0.041	0.334	0.345

Notes: Dependent variables are the child's weight-for-age z score (columns 1, 2, 5, 6, 9 and 10), or weight in kilograms (columns 3, 4, 7, 8, 11 and 12). Difference to mean payout refers to each worker's relative position in their treatment group, based on the number of children whose malnutrition status improved at their center relative to other centers in their geographic location and treatment arm as defined by equations (1), (2) in the main text. Columns with odd numbers use outcomes measured at Endline I in April 2015, which is 3 months after the contest started and was the basis for incentives paid in May 2015. Even-numbered columns use outcomes at Endline II in July 2015. Coefficients have been multiplied by 1000 for clarity. All other notes are as for Table 2.

For the tests reported in Table 3 we use each worker's difference from the mean level of improvement, and expect a negative coefficient implying that lower performers are more incentivized by proportional payments. Workers who are observed to be top performers, and are therefore more likely to be rewarded under winner-take-all, might have high ability. But they might also have benefited from differences among centers such as local disease outbreaks that affected the children in their care more than the children in other centers. If workers know something ahead of time about their ability and relative circumstances, then we would expect significant heterogeneity in the short run, regarding the outcomes at 3 months for which the worker will be rewarded in the contest. If workers learn about differences during the contest, or their short-run effort is measurable only through longer run changes, then we would expect significant heterogeneity in outcomes at 6 months.

Table 3 presents our heterogeneity tests after 3 months in the odd-numbered columns, and after 6 months in the even-numbered columns. This reveals no significant heterogeneity in the power of incentives within the contest period, but consistent and highly significant effects in the longer run after rewards were paid. The gains from using proportional rewards instead of a winner-take-all prize occur primarily among workers who have lower outcomes relative to others. Payments made proportionally to all workers' improvements, instead of just the top performers, are more effective because they reach those who would otherwise be ignored or even discouraged by their lower rank in a winner-take-all contest.

Results reported in Table 3 are potentially endogenous due to use of the endline results, so we conduct two other checks on heterogeneity in treatment response. First, we consider heterogeneity in terms of the worker's ability and circumstances, using their nutrition quiz scores, education levels and class size, as well as the mother's characteristics in terms of her nutrition quiz scores, education level, and the number of children at home. Results reported in the annex Table A6 confirm that the overall significance of proportional rewards arises through the response of the less advantaged workers (lower quiz scores and larger class sizes, although no significance on worker education) and among the mothers with more ability to respond (higher quiz scores and fewer children at home, although no significance for mother's education and a higher response among illiterate mothers). Then, we follow Chetty et al. (2014) to calculate the value-added scores for each worker based on the two rounds of baseline data, and use that to test for heterogeneity in response to the proportional treatment relative to the winner-take-all contest. Results reported in annex Table A7 also confirm our main finding from Table 3, showing that treatment effects are significant because of high response among those who experienced less success before the contest, and are therefore less likely to respond when the contest offers only one winner-take-all prize.

**Fig. 2:**
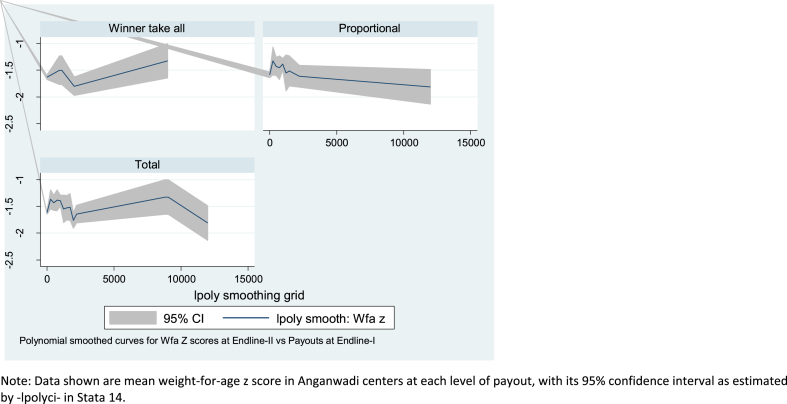
Payout to worker at Endline-1 in Rupees (x axis) and weight for age z score (y axis) at Endline-2, by treatment arm.

Fig. 2 provides a visualization of links between the level of payouts and child weight-for-age z score in the long run, as measured at Endline-2, by treatment arm. As would be expected from the interaction term in Table 3, the slopes differ: for children in centers where workers were offered winner-take-all rewards, weights rose more in the high-payout centers, where there had been more weight gain three months earlier. In contrast, at centers where workers had been offered the proportional payments, there was a more equitable distribution of weight gain, with slightly higher weights in centers that had seen less rise from the baseline to Endline-I.

**Fig. 3:**
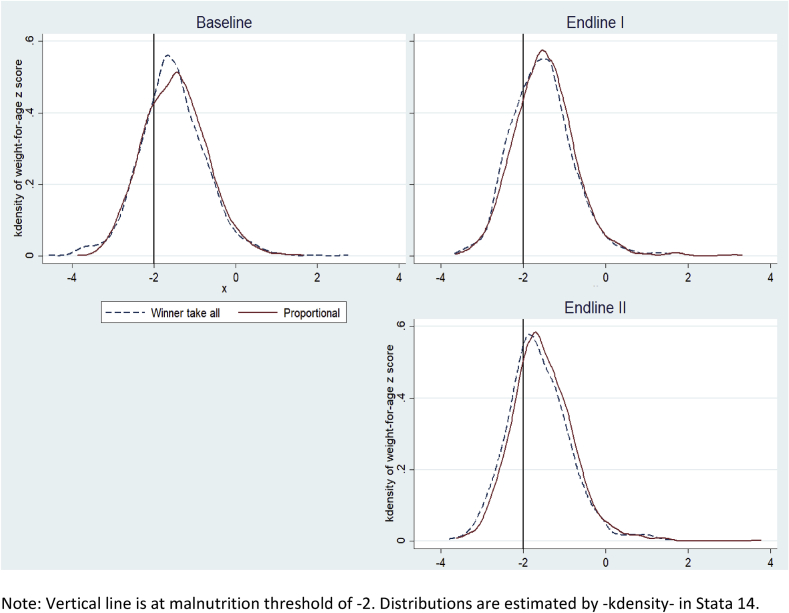
Weight-for-age z scores by type of incentive, before and after treatment.

Fig. 3 provides an alternative kind of visualization, drawing the entire distribution of weight-for-age z scores across all three survey rounds. At baseline (before randomization), the two treatment arms have almost identical fractions of children to the left of the malnutrition cutoff of z = −2. To the right of that cutoff, the winner-take-all arm happens to have somewhat more children just above the threshold up to about −1.5, while the proportional arm happens to have somewhat more children at higher weight levels. By Endline-I and especially Endline-II, the distribution in the proportional treatment arm has shifted to the right of the winner-take-all arm, especially below the cutoff of −2.

Further parametric tests regarding the mechanism by which PRP treatment results in greater improvement than WTA prizes are provided in Table 4, Table 5, using heterogeneity in terms of each child's baseline anthropometric characteristics. If PRP works primarily by spreading incentives even to workers who are less likely to be top performers, its success is likely to be concentrated among more children and centers with more malnutrition. Table 4 provides these tests using weight-for-age z scores, and Table 5 does so with weight-for-age malnutrition status. Both tables reveal that the gains from PRP occur primarily among children who are more underweight, in the sense of being further below the threshold of malnutrition status.

**Table 4:** Mechanism check: heterogeneity of treatment effects on weight-for-age z score, by baseline anthropometry.

	Wfa z (1)	Wfa z (2)	Wfa z (3)	Wfa z (4)	Wfa z (5)	Wfa z (6)	Wfa z (7)	Wfa z (8)
Proportional	−0.507*** (0.147)	−0.0691 (0.0990)	0.0765** (0.0325)	0.100** (0.0443)	−0.530*** (0.158)	−0.0791 (0.105)	0.0681** (0.0345)	0.122*** (0.0470)

Distance from threshold	−0.210*** (0.0589)				−0.225*** (0.0611)			

Distance from threshold*Proportional	0.401*** (0.0959)				0.408*** (0.103)			

Wfa malnutrition prevalence		−0.783*** (0.135)				−0.743*** (0.144)		

Wfa mal. prevalence*Proportional		0.254 (0.232)				0.272 (0.246)		

Change in Wfa z (Baseline I to II)			0.242*** (0.0722)				0.197*** (0.0760)	

Change in Wfa z*Proportional			−0.160 (0.114)				−0.166 (0.121)	

High change in Wfa z (Baseline I to II)				0.0389 (0.0441)				0.0391 (0.0468)

High change in Wfa z*Proportional				−0.0127 (0.0637)				−0.0826 (0.0681)

All controls					X	X	X	X
	2272	2272	2272	2272	1934	1934	1934	1934
adj. R-sq	0.011	0.021	0.008	0.003	0.049	0.056	0.043	0.040

Notes: Dependent variable is the child's weight-for-age z score after 6 months, at Endline II. The first row is the main effect of proportional-reward treatment, and each column tests for heterogeneity in terms of specific anthropometric characteristics at baseline. Columns (1) and (5) use the child's distance to the threshold for improvement in status, columns (2) and (6) use their center's initial prevalence of malnutrition, columns (3) and (7) use the child's pre-trend from baseline I to baseline II, while columns (4) and (8) use an indicator equal 1 if the child had above-average gain in weight-for-age relative to others in their group. All other notes are as for Table 2.

**Table 5:** Mechanism check: heterogeneity of treatment effects on weight-for-age malnutrition status, by baseline anthropometry.

	Wfa mal (1)	Wfa mal (4)	Wfa mal (2)	Wfa mal (3)	Wfa mal (5)	Wfa mal (8)	Wfa mal (6)	Wfa mal (7)
Proportional	0.288*** (0.0941)	0.0300 (0.0633)	−0.0523** (0.0208)	−0.0603** (0.0283)	0.351*** (0.103)	0.0178 (0.0684)	−0.0445** (0.0225)	−0.0638** (0.0307)

Distance from threshold	0.134*** (0.0376)				0.146*** (0.0399)			

Distance from threshold*Proportional	−0.232*** (0.0613)				−0.267*** (0.0670)			

Wfa malnutrition prevalence		0.449*** (0.0862)				0.417*** (0.0943)		

Wfa mal. prevalence*Proportional		−0.136 (0.148)				−0.0912 (0.161)		

Change in Wfa z (Baseline I to II)			−0.0916** (0.0462)				−0.0678 (0.0497)	

Change in Wfa z*Proportional			0.0893 (0.0729)				0.0753 (0.0789)	

High change in Wfa z (Baseline I to II)				0.00245 (0.0282)				−0.0000930 (0.0306)

High change in Wfa z*Proportional				0.0000292 (0.0407)				0.0279 (0.0445)

All controls					X	X	X	X

N	2274	2274	2274	2274	1935	1935	1935	1935
adj. R-sq	0.010	0.017	0.004	0.003	0.029	0.033	0.021	0.020

Notes: Dependent variable is the child's weight-for-age malnutrition status at Endline II. All other notes are as for Table 4.

In Table 4, Table 5, columns 1 and 5 reveal that the main effect of PRP treatment is actually negative at the mean distance of center from threshold, and the positive average treatment effect found in Table 2, Table 3 arises entirely because of greater improvement among centers who have more children who are further below the threshold when the contest starts. Caregivers in the winner-take-all control arm achieve gains primarily among children who are closer to thresholds, and these gains on average are smaller than the gains achieved by caregivers in the proportional-rewards treatment who serve a broader range of children. Columns 2–4 and 6–8 find no other kinds of heterogeneity. As might be expected, children in centers with a higher prevalence of malnutrition have lower weights (columns 2 and 6), but the interaction term with PRP treatment is not significantly positive. Similarly, children that had more positive weight gain from Baseline I to Baseline II had higher weights in Endline II, but again the interaction term with PRP treatment is not significantly positive (columns 3 and 7), even when that relationship is tested using a dichotomous indicator variable (columns 4 and 8).

In summary, our results show that financial incentives for improved outcomes have larger and more significant effects when paid in proportion to improvement, rather than as a winner-take-all prize paid only to top performers. This average treatment effect arises because of improvements among lower-ranked performers and more malnourished children, particularly after the contest ends. These effects of contest design become larger over time, perhaps because their actions during the contest cause later changes in children's weight, or because payouts in the contest influence the workers' later actions through intrinsic motivations and social norms. In previous trials, even unconditional bonuses have elicited some increased effort through gift-exchange mechanisms (e.g. Singh and Masters, 2017). Intrinsic motivations are not directly observable, but some clues may be provided by workers' responses to questions about their level of satisfaction with their own abilities, their work, and their life in general.

## 6 Mechanisms: worker satisfaction and subjective well-being

Table 6 tests for the effect of contest design on workers' satisfaction with their own ability, their work, and their life in general, asked after workers have seen payouts made to them and others. Our hypothesis is that PRP incentives lead to higher satisfaction levels, and does so primarily among the workers with lower payouts whose rank order makes them unlikely to be rewarded in WTA contests. These mechanism tests have less statistical power than our main results, because sample size is limited to the number of workers rather than children, and because worker satisfaction scores have less variance in response to treatment than children's weights. Responses about satisfaction in the worker's own ability and in her work are on a Likert scale (1–5) where 1 means very satisfied and 5 means very dissatisfied, with coefficients reversed so that a positive sign indicates higher satisfaction. For reporting purposes we have reversed the sign, so that a larger score indicates more satisfied. Responses about her satisfaction with life in general are on ladder scale (1–10) where 1 means very unhappy and 10 means very happy.

**Table 6:** Mechanism check: treatment effects on worker's self-reported satisfaction.

	Satisfaction in Ability (1)	Satisfaction in Work (2)	Satisfaction in Life (3)	Satisfaction in Ability (4)	Satisfaction in Work (5)	Satisfaction in Life (6)
Proportional	0.0809 (0.218)	0.379 (0.289)	0.311 (0.422)	0.227 (0.225)	0.406 (0.298)	0.528 (0.434)

Lower absolute payout (×1000)	−0.0456 (0.111)	−0.150 (0.146)	−0.329 (0.216)			

Lower abs. payout*Proportional (×1000)	0.330** (0.138)	0.0866 (0.182)	0.514* (0.268)			

Lower relative payout (×1000)				−0.0727 (0.109)	−0.128 (0.143)	−0.370* (0.211)

Lower rel. payout*Proportional (×1000)				0.349** (0.137)	0.0527 (0.180)	0.518* (0.265)

Constant	1.493*** (0.153)	1.854*** (0.205)	7.173*** (0.298)	1.521*** (0.156)	1.915*** (0.208)	7.013*** (0.302)

N	83	82	84	83	82	84
adj. R-sq	0.101	0.006	0.016	0.096	0.004	0.018

Notes: Dependent variable is self-reported at Endline II, 6 months after incentives were offered and 3 months after payouts were made. Workers' satisfaction with their own ability and in their work was reported on a 1–5 scale, where 1 means very satisfied and 5 means very dissatisfied. For reporting purposes we have reversed the sign, so that a larger score is more satisfied. Workers' satisfaction with their life in general was reported on a 1–10 scale, where 1 means very unhappy and 10 means very happy. Columns 1–3 use the cash value of the worker's payout, while columns 4–6 use the worker's payout relative to the mean paid in their treatment group (their treatment arm in their geographic location). *Significant at 10%, **Significant at 5%, *** Significant at 1%.

The first row of Table 6 reveals that the main effect of PRP incentives on an ‘average payout worker’ is positive but always insignificant. Columns 1–3 control for the worker's absolute level of payout, and columns 4–6 control for their payout relative to the mean of their treatment group. In both cases a lower payout implies a lower rank order, and hence smaller likelihood of success in a WTA contest. We find that the main effect of lower payout is negative but insignificant in all but column 6, and the interaction term are significant at p = 0.05 only for workers' own ability and at p = 0.10 for worker's life satisfaction. This provides only suggestive evidence that PRP incentives leave lower-ranked workers more satisfied with their abilities and with life, implying less discouragement as compared to a WTA contest. This is consistent with previous studies that find lower satisfaction among losers of a WTA contest.

## 7 Mechanisms: worker effort as reported by children's mothers

Table 7 tests for the effect of contest design on mothers' perception of worker effort. This is measured by interviews with the mothers of children in each center in our Endline-II survey regarding worker efforts in the previous month. We are therefore comparing responses about efforts made after the end of incentives, between workers who received PRP rather than WTA incentives. Questions ask for the mother's recall of: (1) the number of home visits made by the worker, (2) how many times the worker discussed the child's development at the center or elsewhere, (3) the number of times group meetings were organized at the Anganwadi center and (4) the number of other meetings convened by the worker. We also ask mothers about the content of communication from the workers, which are coded as discrete indicators whether the worker gave them any advice in the previous month on (1) nutrition and diet, (2) hygiene, (3) medicine, or if they (4) showed them their child's growth chart, or (5) scared them about consequences of malnutrition.

**Table 7:** Mechanism check: treatment effects on worker effort as reported by mothers.

	Quantity of effort (number of events)				Topic of communication				
	Home visits (1)	Talks about child (2)	Group AWC meetings (3)	Other meetings (4)	Nutrition (5)	Hygiene (6)	Medicine (7)	Growth chart (8)	Scare (9)
Proportional	0.36 (1.065)	0.696 (1.026)	0.329 (0.698)	0.21 (0.879)	0.00523 (0.0224)	0.0771 (0.0635)	0.0598 (0.073)	0.00511 (0.0689)	0.0114 (0.0622)

Child and mother-level controls	X	X	X	X	X	X	X	X	X
Worker controls	X	X	X	X	X	X	X	X	X

N	1718	1852	1518	1472	1860	1860	1860	1860	1860
Adj. R-sq	0.054	0.107	0.044	0.151	0.028	0.245	0.038	0.061	0.186

Notes: The dependent variable for each column is the mother's responses to survey questions at Endline II, about the number of times in the previous month that the Anganwadi worker: (1) visited their home, (2) talked about the child, (3) held a group meeting at the Anganwadi center, or (4) met with the mother at any other location, and whether the Anganwadi worker ever discussed: (5) nutritional values of different foods, (6) hygienic practices to avoid illness, (7) medical services to treat illness, (8) growth monitoring of the child's height for age, or (9) scary consequences of malnutrition. All other notes are as for Table 2.

Results presented in Table 7 show positive point estimates but no statistical significance for any of these effort measures in the proportional rather than winner-take-all treatment. This provides only suggestive evidence that efforts were higher in the proportional arm, which could be due to not having sufficiently accurate measures of what workers actually did to achieve the weight gains we observe. Future studies could focus on improving measurement of each kind of effort, so as to identify the changes made by the workers whose response to additional incentives is most successful.

## 8 Conclusion

This study presents results of a randomized trial comparing two kinds of financial incentives to improve child nutrition outcomes at ICDS daycare centers in Chandigarh, India. Such incentives are increasingly being introduced in education, health and other services to improve outcomes that are measurable but not sufficiently rewarded by workers' current salaries and other employment arrangements. The incentives offered in this study use a fixed budget in a time-limited contest designed to elicit additional efforts, reveal best practices and pay bonuses that reward success.

Our trial compares a winner-take-all prize paid to the best performer, which is by far the most widely-used contest design, against an alternative approach in which the same information is used to divide the same funds proportionally among all successful workers based on their share of measured gains during the contest period. The two treatments offered in this experiment use the same budget and identical information, differing only in whether payments are made on a winner-take-all (WTA) or proportional (PRP) basis.

The comparison we offer between two otherwise identical incentive schemes is intended to complement the many previous trials that introduce financial rewards relative to a status-quo control or other arms in which workers receive different amounts of money and information. This paper aims specifically to complement Singh and Masters (2017), which compares the introduction of piece-rate incentives to an unconditional fixed bonus among otherwise similar Anganwadi workers in other areas of Chandigarh. That paper found that piece-rate incentives for each increment of success led to higher performance than the fixed bonus, but also ended up paying more money to workers than the fixed bonus. Implementing piece rates is challenging in part because development agencies typically operate on fixed annual budgets. The incentive schemes introduced in this paper involve payments whose timing and upper limit of total cost is entirely predictable, differing only in how they are divided among workers.

Payments offered in this trial are set at 600 Rs per worker, which amounts to a roughly 5% increment above workers' monthly salary over the 3-month period of the contest. In Chandigarh, like other parts of India, each Anganwadi workers operates her own ICDS center that typically serves about 25 preschool children every day. Centers are grouped in geographic clusters of varying sizes, depending primarily on population density of children in the low-income families that the ICDS program is designed to serve. Payouts are awarded based on each worker's performance relative to others in their neighborhood, who presumably share information and face common shocks as well as other unobservable characteristics.

The bonus paid to each worker is based on the number of children at their center whose malnutrition status improves, minus any declines below the threshold weight-for-age z score of −2. Anganwadi workers were individually randomized into either a traditional WTA contest, or the more novel PRP treatment. With winner-take-all incentives, the highest-ranked worker in each cluster receives the entire reward budgeted for their cluster. When allocated proportionally, every worker with net improvements receives a fraction of that award, based on their share of all net improvements realized in their cluster. Our hypothesis is that proportional rewards would be more encouraging than winner-take-all prizes for workers whose results are unlikely to be top-ranked, thereby eliciting more total effort from the entire cluster, as suggested by theoretical models and laboratory results such as Cason et al. (2010, 2018) and evidence from athletic competitions such as Brown (2011).

Results reveal consistently beneficial (although imprecisely measured) effects of using proportional awards instead of the winner-take-all prize. The average treatment effect over our entire sample is a decline of 4.3 percentage points in the prevalence of malnutrition over the 3 month contest period, increasing to 5.9 points at 6 months after the contest has ended. Those improvements in the ICDS program's nutritional objective translate to improvements in average weight-for-age z scores of 0.071 standard deviation units at 3 months, rising to 0.095 at 6 months. Children in the care of workers with proportional incentives improved significantly more, especially after the contest ended, with persistent effects that could operate either through momentum in child growth or sustained changes in worker behavior. Mechanism tests reveal that, after the contest is over, the workers who were randomized into our proportional-incentive arm and were not highly ranked had higher self-reported satisfaction in life and also in ability. This parallels the greater gains observed among children in their care. Workers' efforts as reported by mothers have positive but not statistically significant point estimates.

The internal validity of our results is strengthened by individual randomization into treatment groups within neighborhoods that share common information, management styles and other unobservable characteristics. We found no statistically significant differences at baseline between the treatment arms in our variables of interest, and also tested for possible artefactual effects of our study design on placebo outcomes (weight changes prior to intervention). The data we report include child outcomes and worker satisfaction several months after the incentives were announced and paid; further work could include even longer-term outcomes, diffusion of best practices from workers who received bonuses, and selection into employment where effort is more likely to be rewarded.

Replication to test external validity is facilitated by the modular structure, simple design and low cost of our study. We show that both PRP and WTA contests can be introduced into groups as small as just two workers, up to the largest group in our trial which had 19 workers. Either kind of incentive could be scaled up by local authorities across India's 1.3 million ICDS centers, or introduced to other service-delivery agencies that also aim to improve a measurable outcome. In our trial, implementation and data collection was undertaken by a local NGO, which could be done elsewhere through contracts with independent survey firms to avoid favoritism or self-interest in the measurement process. Budgeting and administration is facilitated by making the bonus pool just large enough to attract workers' attention, in this case about 5% of monthly payroll offered as a one-time incentive on a fixed timeline.

One-time bonuses like those tested in this trial are often used to complement fixed salaries in settings where outcomes can be measured but other types of pay for performance are not desirable. We find that such contests elicit better results when payments are disbursed in proportion to measured gains, especially over the longer run after the contest has ended. The standard approach of a winner-take-all-prize is more highly powered, concentrating the available funds to motivate top performers, but we find that cumulative total effort of all workers is lower because winner-take-all contests provide no incentive for lower-ranked workers to improve. Proportional incentives reward every increment of success, from every worker, including those whose outcomes are initially low. Delivery of other public services in education, health and other sectors might benefit from this approach, offering opportunities for further testing and eventual scale-up for the type of financial incentive introduced in this trial.
