# Supplementary material for: Performance bonuses in the public sector: Winner-take-all prizes versus proportional payments to reduce child malnutrition in India
Source: J Dev Econ. 2020 Sep;146:102295. doi: 10.1016/j.jdeveco.2018.10.003 (PMC7457730; doi:10.1016/j.jdeveco.2018.10.003)
Supplement: Multimedia component 2 [file mmc2.docx]

**ANNEX OF SUPPLEMENTAL INFORMATION**

**Figure A1. Timeline of the experiment**


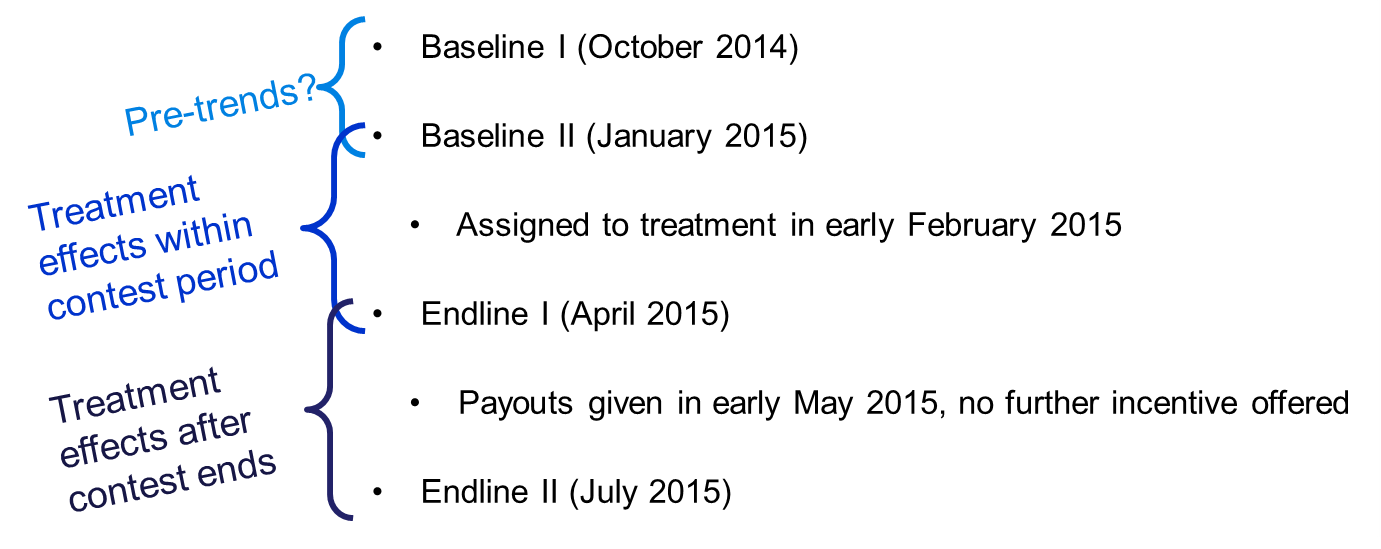


Figure A2. Histogram of baseline child ages and weights

Panel A: Age

(in months)

Panel A: Weight

(in kilograms)

Note: Data shown are measured at Baseline II.

Figure A3. Scatterplot of change in weight for age z score by its baseline level

Note: Data shown are changes from Baseline II to Endline I, by level in Baseline II

**Figure A4. Histogram of payouts by treatment arm**

**Table A1. Number of workers by treatment group**

|  | |  | | Treatment Arm | | |
| --- | --- | --- | --- | --- | --- | --- |
| Location | | Total | | WTA | | PRP |
| Adarsh | | 3 | | **3** | | ***** |
| Badheri | | 4 | | **2** | | **2** |
| Buterla | | 4 | | **2** | | **2** |
| Dhanas | | 38 | | **19** | | **19** |
| Ramdarbar | | 29 | | **14** | | **15** |
| Sector 52 | | 7 | | **3** | | **4** |
|  | | 85 | | 43 | | 42 |
| Note: The Adarsh neighborhood had only 3 centers, so could be allocated only one type of contest which was randomly picked to be WTA. | | | | | | |

| **Table A2. Number of children by treatment group** | | | |
| --- | --- | --- | --- |
|  |  | Treatment Arm | |
| Location | Total | WTA | PRP |
| Adarsh | 121 | **121** | ***** |
| Badheri | 132 | **62** | **70** |
| Buterla | 93 | **56** | **37** |
| Dhanas | 973 | **505** | **468** |
| Ramdarbar | 777 | **374** | **403** |
| Sector 52 | 244 | **107** | **137** |
|  | 2,340 | 1,225 | 1,115 |
| Note: Data shown is number surveyed at first endline, for short-term effects after 3 months. | | | |

Tables A2.2 and A2.3 highlight the attrition rates in the two groups at every round and also that the attrition was random across treatment arms. Attrition levels are high but consistent across the units because children are often absent from the center (either because child stays at home but mother comes to collects food from center, parents migrate or child transfers to another school). Thus, it is difficult to sample the same set of children again in the center across two rounds. These attrition levels are in line with other studies based in the Anganwadi setting.

| **Table A2.2: Attrition rates** | | | | | | | | | | | |
| --- | --- | --- | --- | --- | --- | --- | --- | --- | --- | --- | --- |
|  | | | | | **Proportional** | | **Winner-take-all** | | | **Total** | |
| **Baseline** | children weighed | | | | 1115 | | 1225 | | | 2340 | |
| **Endline-I** | children re-weighed | | | | 831 | | 903 | | | 1734 | |
|  | **Attrition in children weighed from Baseline** | | | | 25% | | 26% | | | 26% | |
| **Endline-II** | children re-weighed from Baseline | | | | 663 | | 729 | | | 1392 | |
|  | **Attrition in children weighed from Endline-I** | | | | 20% | | 19% | | | 20% | |
| Notes: Attrition is not significantly different between the treatments at Endline-I or at Endline-II. The p-values for the differences in attrition at Endline-I and Endline-II are 0.66 and 0.92 respectively. | | | | | | | | | | | |
|  |  |  |  |  |  |  |  |  |  |  |  |
| **Table A2.3: Random Attrition across groups for main indicators** | | | | | | | | | | |  |
|  | | At baseline across non-attrited children in Endline-I | | | | At baseline across non-attrited children in all rounds | | | | |  |
|  | | Weight | Wfa z | Wfa mal | | Weight | | Wfa z | Wfa mal | |  |
| Proportional | | 13.73 | -1.45 | 0.26 | | 13.67 | | -1.45 | 0.25 | |  |
| Winner-take-all | | 13.70 | -1.52 | 0.27 | | 13.59 | | -1.51 | 0.25 | |  |
| Difference | | 0.03 | 0.07 | -0.01 | | 0.08 | | 0.06 | 0 | |  |
| p-value of differences for non-attrited children | | 0.44 | 0.38 | 0.24 | | 0.91 | | 0.77 | 0.69 | |  |

**Table A3. Budgeted and actual payouts by treatment arm**

|  | WTA | PRP |
| --- | --- | --- |
| **Budgeted payouts (ex-ante)** |  |  |
| Budgeted payout per worker | 600 | 600 |
| Total number of workers | 43 | 42 |
| Total number of slum clusters | 6 | 5 |
|  |  |  |
| **Actual payouts (ex-post)** |  |  |
| Average payout per worker | 279* | 600 |
| Average payout per payee | 4000 | 1575 |
| Number of workers receiving payouts | 3* | 16 |
| Number of clusters without payouts | 3* | 0 |

Note: In the WTA arm, 3 of 6 clusters had no net improvement, hence no payout.

| **Table A4. Placebo test for artefactual effects before intervention** | | | | | | |
| --- | --- | --- | --- | --- | --- | --- |
|  |  |  |  |  |  |  |
|  | Weight | Wfa z | Weight | Wfa z | Weight | Wfa z |
|  | (1) | (2) | (3) | (4) | (5) | (6) |
| Proportional | -0.0737 | -0.0423 | -0.0663 | -0.0376 | -0.0364 | -0.0222 |
|  | (0.121) | (0.0563) | (0.138) | (0.0640) | (0.141) | (0.0649) |
|  |  |  |  |  |  |  |
| Child and mother-level controls |  |  | X | X | X | X |
| Worker controls |  |  |  |  | X | X |
| N | 1760 | 1751 | 1288 | 1287 | 1288 | 1287 |
| adj. R-sq | 0.000 | 0.001 | 0.000 | -0.001 | 0.019 | 0.018 |
| Notes: Results shown use change between two rounds of data collected before intervention, from Baseline I (October 2014) to Baseline-II (January 2015). Baseline I was collected only to serve as a practice round of data collection and to check for parallel trends before intervention, while Baseline II served as the starting point for financial incentives that were offered starting immediate after these data were collected and workers were randomized into treatment. All other notes are as for Table 2. | | | | | | |

**Table A6. Heterogeneity by class size, worker characteristics, mother characteristics and household size**

Note: results shown are for long-run impacts measured at Endline II.

**Table A7. Heterogeneity by level of provider value added in baseline rounds**

|  | Low improvement (below median) | | | High improvement (above median) | | |
| --- | --- | --- | --- | --- | --- | --- |
|  | Weight | Wfa z | Wfa mal | Weight | Wfa z | Wfa mal |
|  |  |  |  |  |  |  |
| Proportional | 0.360** | 0.166* | -0.139*** | 0.0861 | 0.0462 | 0.00757 |
|  | (0.175) | (0.0832) | (0.0439) | (0.159) | (0.0581) | (0.0348) |
| N | 1085 | 1062 | 1062 | 1240 | 1210 | 1210 |

Note: results shown are for long-run impacts measured at Endline II.
